# CD169^+^ Skin Macrophages Function as a Specialized Subpopulation in Promoting Psoriasis-like Skin Disease in Mice

**DOI:** 10.3390/ijms25115705

**Published:** 2024-05-24

**Authors:** Mengyao Li, Wenjing Yu, Zhiduo Liu, Siming Liu

**Affiliations:** Department of Immunology and Microbiology, Shanghai Institute of Immunology, Shanghai Jiao Tong University School of Medicine, Shanghai 200025, China; limengyao28@sjtu.edu.cn (M.L.); wenjingyu@shsmu.edu.cn (W.Y.)

**Keywords:** CD169, CD169-DTR mice, IMQ-induced psoriasis mouse model, Th17 cells

## Abstract

Skin macrophages are critical to maintain and restore skin homeostasis. They serve as major producers of cytokines and chemokines in the skin, participating in diverse biological processes such as wound healing and psoriasis. The heterogeneity and functional diversity of macrophage subpopulations endow them with multifaceted roles in psoriasis development. A distinct subpopulation of skin macrophages, characterized by high expression of CD169, has been reported to exist in both mouse and human skin. However, its role in psoriasis remains unknown. Here, we report that CD169^+^ macrophages exhibit increased abundance in imiquimod (IMQ) induced psoriasis-like skin lesions. Specific depletion of CD169^+^ macrophages in CD169-ditheria toxin receptor (CD169-DTR) mice inhibits IMQ-induced psoriasis, resulting in milder symptoms, diminished proinflammatory cytokine levels and reduced proportion of Th17 cells within the skin lesions. Furthermore, transcriptomic analysis uncovers enhanced activity in CD169^+^ macrophages when compared with CD169^−^ macrophages, characterized by upregulated genes that are associated with cell activation and cell metabolism. Mechanistically, CD169^+^ macrophages isolated from IMQ-induced skin lesions produce more proinflammatory cytokines and exhibit enhanced ability to promote Th17 cell differentiation in vitro. Collectively, our findings highlight the crucial involvement of CD169^+^ macrophages in psoriasis development and offer novel insights into the heterogeneity of skin macrophages in the context of psoriasis.

## 1. Introduction

Psoriasis, a prevalent complex inflammatory skin disease, afflicts approximately 2–3% of the global population [1,2]. The most common clinical manifestation of psoriasis is plaque psoriasis, characterized by well-defined, erythematous areas of skin covered with silvery scales. The disease can develop at any age and exhibits no gender preference [3,4]. In addition to genetic factors, non-genetic triggers, including stress, trauma, infection, obesity, medication and weather, can also precipitate the onset of psoriasis [5]. However, there is no definitive cure for psoriasis yet. While clinical treatments can ameliorate symptoms of psoriasis, relapses are common and the chronic nature of the disease severely impairs patients’ quality of life [6].

Psoriasis is pathologically characterized by the aberrant proliferation and differentiation of keratinocytes, as well as the infiltration of numerous immune cells within the skin lesions [7]. Over the past few decades, an extensive body of research has revealed the involvement of a variety of immune cells and immune-related pathways in the pathogenesis of psoriasis. Among these pathways, the IL-23/IL-17 axis emerges as a pivotal driver of psoriasis [8]. The prevailing paradigm in psoriasis pathogenesis is that autoantigens, specifically self-nucleotides and antimicrobial peptides derived from keratinocytes, activate the resident dermal dendritic cells. Subsequently, these activated dendritic cells initiate the production of pro-inflammatory cytokines, such as IL-23, IL-12, and TNF-α, which in turn induce T cell differentiation [7,9]. IL-23, a heterodimeric cytokine consisting of two subunits, IL-23p19 (an IL-23 specific subunit) and IL-12p40 (a shared subunit with IL-12), is regarded as a master regulator in the differentiation of IL-17-producing T cells and IL-17 secretion [10]. IL-17, predominantly secreted by Th17 and γδ T cells, exerts a critical function in stimulating the mitogen-activated protein kinase (MAPK) and NF-κB signaling pathways [11]. Consequently, IL-17 directly accelerates the proliferation of keratinocytes and elicits a feedforward inflammatory response by triggering cytokine and chemokine production [12,13]. Multiple therapeutic approaches targeting IL-23 or IL-17 have garnered clinical approval, thereby further underscoring the importance of the IL-23/IL-17 axis in the pathogenesis of psoriasis [14].

Macrophages, the predominant resident immune cell population in the skin, exhibit diverse origins [15]. While dermal macrophages are primarily derived from yolk sacs and fetal liver, a minor fraction is continuously replenished by bone marrow monocytes [16]. As crucial components of the innate immune system, macrophages sense and respond to pathogens and environmental cues, thereby contributing significantly to the maintenance of skin homeostasis. In contrast with T cells, studies that focus on the role of macrophages in psoriasis remain limited. However, prior studies have demonstrated an increased presence of dermal macrophages in psoriatic lesions when compared with normal skin [17,18]. Significantly, macrophages are recognized as a major source of TNF-α in psoriatic lesions [18]. Furthermore, murine studies have demonstrated that clodronate liposome-mediated depletion of macrophages alleviates the severity of psoriasiform skin inflammation [19,20]. These findings indicate the indispensable role played by dermal macrophages in the pathogenesis of psoriasis.

Macrophages are a highly heterogeneous cell population, encompassing distinct subpopulations with unique transcriptional programs and functions. Over the past three decades, a distinctive subset of macrophages, CD169^+^ macrophages, has garnered significant attention. CD169, also known as Siglec1, is a sialic acid-binding immunoglobulin-like lectin expressed primarily on myeloid cells. CD169^+^ macrophages are distributed across various tissues and have been shown to play a significant role in numerous biological processes and disease [21,22]. For instance, the infiltrating CD169^+^ macrophages within gliomas promote antitumor immune responses by producing pro-inflammatory chemokines and phagocytosing apoptotic glioma cells [23]. Moreover, CD169^+^ macrophages promote erythropoiesis in the bone marrow and are critical for erythropoietic recovery from stress [24]. Intriguingly, CD169^+^ macrophages have also been identified in the dermis of both mouse and human skin [25]. However, their involvement in psoriasis remains to be elucidated.

In this study, we investigated the role of CD169^+^ macrophages in psoriasis pathogenesis by utilizing the IMQ-induced psoriasis mouse model. Our findings reveal a progressive increase in CD169^+^ macrophages in the skin, accompanied by changes in their localization, during psoriasis development. Selective depletion of CD169^+^ macrophages inCD169-DTR mice has been shown to lead to the amelioration of psoriasis symptoms, accompanied by reduced levels of proinflammatory cytokines and a lower proportion of Th17 cells in the skin lesions. Furthermore, we identified a distinct gene signature that is specific to CD169^+^ skin macrophages treated with IMQ. We also conducted an ex vivo coculture experiment and have proved that CD169^+^ skin macrophages isolated from psoriasis lesions facilitated the differentiation of Th17 cells. Overall, our data contribute to a comprehensive understanding of skin macrophage heterogeneity and highlight the significant role of CD169^+^ macrophages in psoriasis pathogenesis.

## 2. Results

### 2.1. CD169^+^ Macrophages Are More Abundant and Appear near Epidermis in Psoriasis-like Lesions

**Figure 1:**
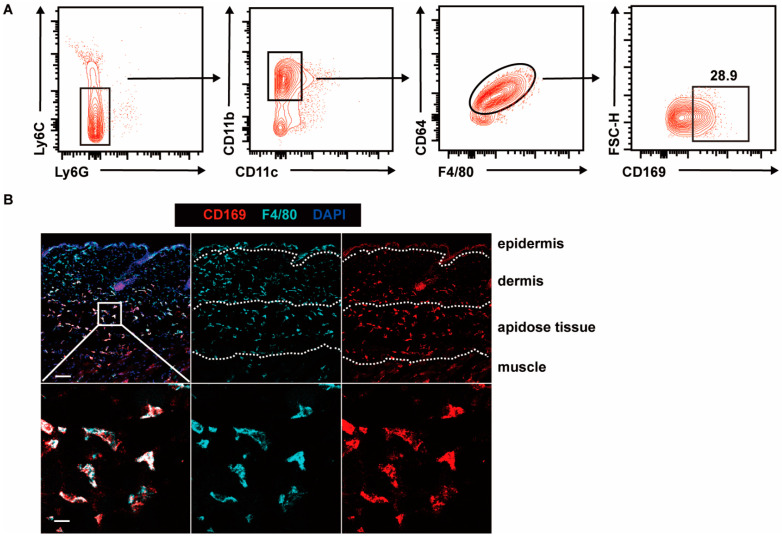
The distribution of CD169^+^ macrophages in mouse skin. (**A**) Representative flow cytometry plots show the percentage of CD169^+^ macrophages in WT mice skin. The cells were gated from Live CD45^+^ cells (*n* = 3). (**B**) Representative immunofluorescence staining images show the distribution of CD169^+^ macrophages in WT mice skin (red: CD169, cyan: F4/80, blue: DAPI). Scale bar: 100 μm (**top**) or 20 μm (**bottom**). Data are derived from at least two independent experiments.

We initially examined the proportion and localization of CD169^+^ macrophages in murine skin using flow cytometry and immunofluorescence staining. Consistent with previous reports [25,26], a distinct subset of skin macrophages (CD45^+^Gr1^−^CD11c^−^CD11b^+^CD64^+^F4/80^+^) showed high expression of CD169. These CD169^+^ macrophages represented approximately 28% of the skin macrophage population, as determined by flow cytometry (Figure 1A). Notably, these CD169^+^ macrophages were predominantly situated in the lower dermis and dermal white adipose tissue (Figure 1B). 

**Figure 2:**
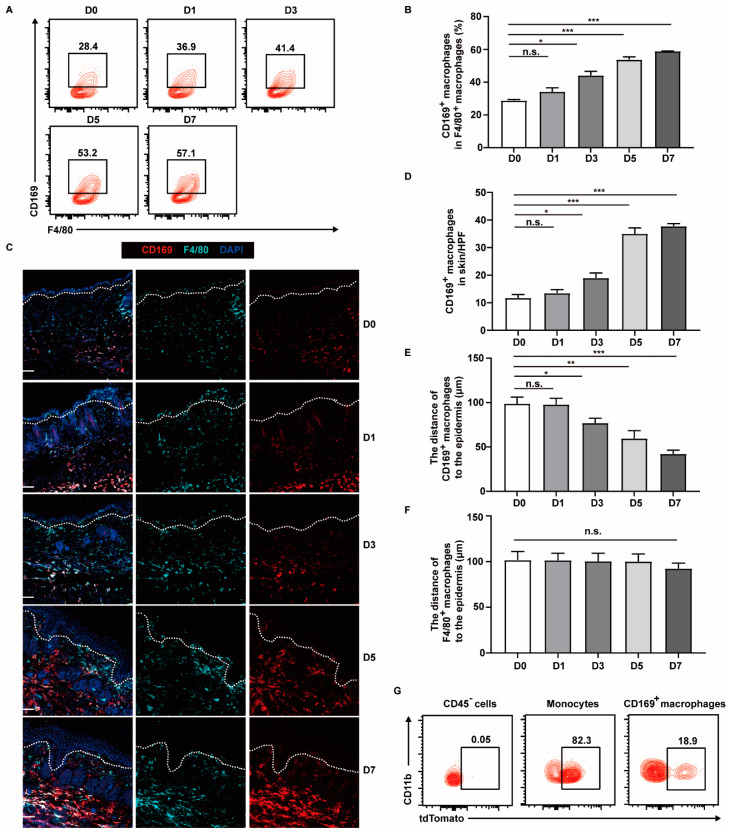
The dynamics of CD169^+^ skin macrophages’ quantity and distribution during the development of IMQ−induced psoriasis. (**A**) Representative flow cytometry plots show the percentage of CD169^+^ macrophages in the skin in WT mice during IMQ treatment (day 0–7 [D0–7]). (**B**) Quantification of the percentage of CD169^+^ macrophages in F4/80^+^ skin macrophages detected by flow cytometry during IMQ treatment (*n* = 3 each time point). (**C**) Representative immunofluorescence staining images show the number and the location of CD169^+^ macrophages in the skin during IMQ treatment in WT mice (red: CD169, cyan: F4/80, blue: DAPI). Scale bar: 100 μm. (**D**) Quantification of the numbers of CD169^+^ macrophages as in (**C**) (*n* = 3 each time point). (**E**,**F**) Quantification of the average distance of CD169^+^ macrophages (**E**) and F4/80^+^ macrophages (**F**) to the epidermis on the days indicated during IMQ application (*n* = 3 each time point). (**G**) Representative flow cytometry plots show the percentage of tdTomato^+^ cells in each group. The cells were gated from live cells. Data represent at least two independent experiments. *** *p* < 0.001, ** *p* < 0.01, * *p* < 0.05, n.s. no significance (Student’s *t*-test).

To explore the dynamics of CD169^+^ macrophages during the progression of psoriasis, we utilized a well-established murine model in which IMQ, a toll-like receptor 7/8 agonist, was topically applied for six consecutive days. This model replicates several key features of human plaque psoriasis, such as skin erythema, thickening, scaling, excessive keratinocyte proliferation and immune cell infiltration [27]. We obtained dorsal skin samples from mice before (day 0) and after IMQ application (day 1, 3, 5, 7) and assessed the abundance and distribution of CD169^+^ skin macrophages. We observed that the percentage of CD169^+^ macrophages in F4/80^+^ cells gradually increased from 28.4% (±0.53%) to 57.6% (±0.42%) during psoriasis development (Figure 2A,B). Similarly, the immunofluorescence staining showed a progressive increase in the number of CD169^+^ macrophages, with a 3-fold increase at day 5 and day 7 compared with day 0 (Figure 2C,D). Meanwhile, we found that these CD169^+^ macrophages gradually appeared in the upper dermis. Following IMQ treatment, their distance to the epidermis, where keratinocytes are located, decreased, while the localization of the overall population of F4/80^+^ cells remained unchanged (Figure 2C,E,F). These findings suggest the potential involvement of CD169^+^ skin macrophages in the pathogenesis of psoriasis. 

We speculated that the increased CD169^+^ skin macrophages are derived from monocytes, as these CD169^+^ skin macrophages showed extremely low proliferation abilities, as indicated by their poor colocalization of the cell proliferation marker Ki-67 (Figure S1). Therefore, we used *Ms4a3^CreERT2^*-*Rosa26^tdTomato^* mice, which can efficiently label the vast majority of monocytes and their progenies with tdTomato after tamoxifen injection [28], to check the origin of the increased CD169^+^ skin macrophages. The mice were intraperitoneally injected with tamoxifen for three successive days prior to IMQ application and were given tamoxifen every other day during IMQ application. They were then analyzed on day 6 post IMQ treatment. As expected, almost 82% of monocytes were tdTomato^+^, while nearly no CD45^−^ cells were labeled, serving as a negative control. Interestingly, approximately 18% of CD169^+^ skin macrophages were tdTomato^+^ on day 5 after IMQ treatment, proving that monocytes contribute to the increased CD169^+^ skin macrophage pool during psoriasis development (Figure 2G).

### 2.2. Depletion of CD169^+^ Macrophages Inhibits IMQ-Induced Psoriasis

**Figure 3:**
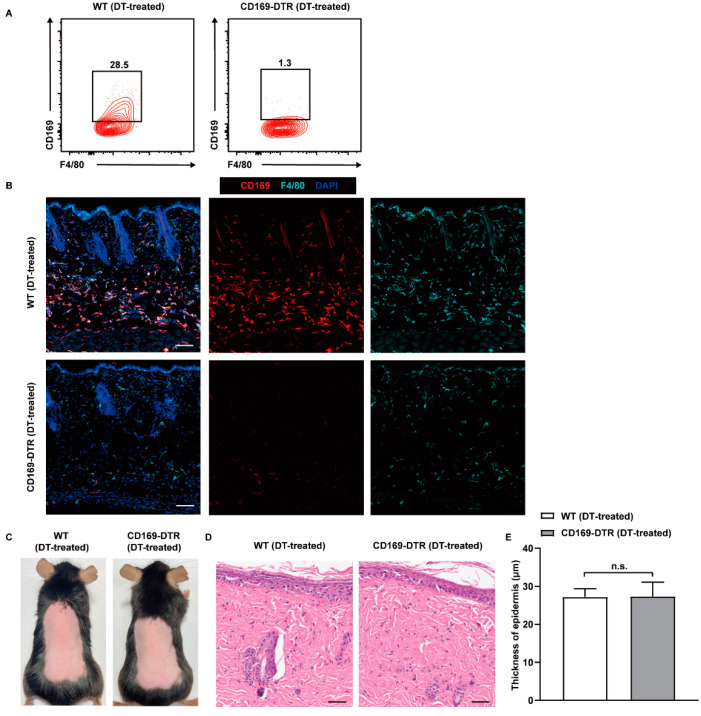
Depletion of CD169^+^ skin macrophages alone does not cause skin abnormalities. WT and CD169-DTR mice were injected intraperitoneally with DT (20 ng/g body weight) every other day for one week. (**A**) Representative flow cytometry plots show the percentage of CD169^+^ skin macrophages in WT and CD169-DTR mice 24 h after DT treatment. The cells were gated from live CD45^+^Gr1^−^CD11c^−^CD11b^+^CD64^+^F4/80^+^ cells (*n* = 3). (**B**) Representative immunofluorescence staining of CD169^+^ macrophages in the skin of WT and CD169-DTR mice 24 h after DT treatment (red: CD169, cyan: F4/80, blue: DAPI). Scale bar: 100 μm. (**C**,**D**) Representative gross appearance and H&E staining of the back skin of WT and CD169-DTR mice on day 7. Scale bar: 100 μm in (**D**). (**E**) Epidermal thickness of WT and CD169-DTR mice on day 7 (*n* = 4). Data represent at least two independent experiments. n.s. no significance (Student’s *t*-test).

To investigate the role of CD169^+^ macrophages in psoriasis, we utilized CD169-DTR mice, which express the knocked-in human diphtheria toxin receptor (DTR) under the regulatory control of the CD169 promoter, enabling selective depletion of CD169^+^ macrophages after administration of diphtheria toxin (DT) to the mice. We first evaluated the efficiency of depletion in CD169-DTR mice. Flow cytometric analysis showed a nearly 98% reduction in CD169^+^ macrophages in the skin 24 h after a single DT injection (Figure 3A). Immunofluorescence staining further validated the result, as a significant decline in CD169 signal was observed in the skin of CD169-DTR mice compared with DT-treated WT mice, while a population of F4/80 single positive cells remained evenly distributed throughout the skin (Figure 3B). These results unequivocally demonstrate the successful and complete depletion of CD169^+^ skin macrophages achieved through DT treatment in CD169-DTR mice.

The skin, functioning as a vital barrier organ, is in direct contact with the external environment and serves as the primary line of defense. Its homeostasis is tightly regulated through intricate crosstalk among epithelial, stromal and immune cells [29]. We wondered whether depletion of CD169^+^ macrophages could directly impact the steady state of the skin. To this end, we continuously depleted CD169^+^ skin macrophages in CD169-DTR mice by administering DT every 48 h for one week. We observed no discernible alterations in the skin appearance of CD169-DTR mice when compared with DT-treated WT mice (Figure 3C). Additionally, the hematoxylin–eosin (H&E) staining of the skin samples revealed similar epidermal thickness and immune cell infiltration between the two groups, demonstrating that simple depletion of CD169^+^ macrophages is insufficient to induce skin abnormalities (Figure 3D,E).

**Figure 4:**
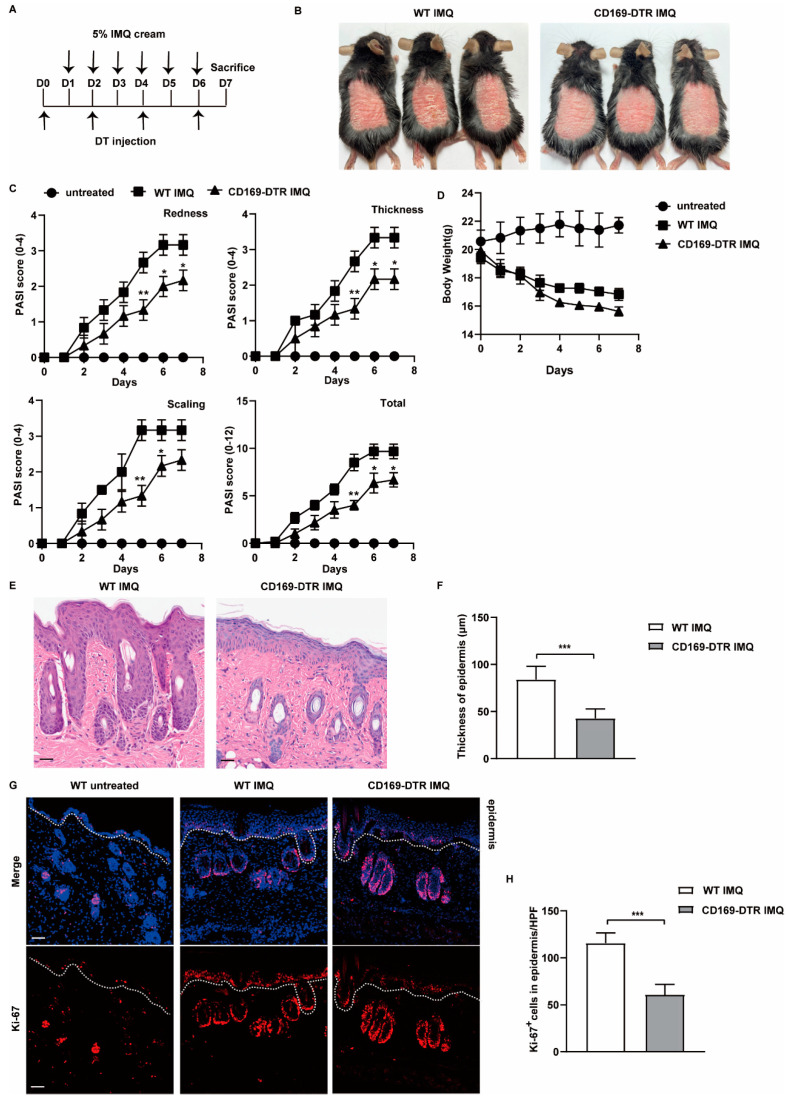
Depletion of CD169^+^ macrophages alleviate the symptoms of psoriasis in the IMQ-induced mouse model. WT and CD169-DTR mice were injected intraperitoneally with DT (20 ng/g body weight) on days 0, 2, 4 and 6. From day 1 to day 6, IMQ was applied daily to the shaved back skin of the mice. The mice were sacrificed on day 7. (**A**) Schematic diagram of the experimental design. (**B**) Representative gross appearance of the back skin of WT and CD169-DTR mice on day 7. (**C**) PASI scores of the back skin were recorded daily (*n* = 3–4). (**D**) Body weight was recorded daily (*n* = 3–4). (**E**) Representative H&E staining of the back skin of WT and CD169-DTR mice on day 7. (**F**) Epidermal thickness of WT and CD169-DTR mice on day 7 (*n* = 3–4). Scale bar: 100 μm. (**G**) Representative immunofluorescence staining of Ki-67 in the skin of WT and CD169-DTR mice on day 7 (red: Ki-67, blue: DAPI, *n* = 3–4). Scale bar: 100 μm. (**H**) Quantification of Ki-67 positive cells in the epidermis in (**G**). Data represent at least two independent experiments. *** *p* < 0.001, ** *p* < 0.01, * *p* < 0.05 (Student’s *t*-test).

Next, we established the IMQ-induced mouse model utilizing both WT and CD169-DTR mice. As shown in the experimental workflow, both groups of mice received daily topical IMQ treatment on the shaved back skin for six consecutive days, with DT injections administered prior to and during IMQ application (Figure 4A). Both groups of mice exhibited erythema, scaling and skin thickening. However, these symptoms appeared less severe in CD169-DTR mice on day 7 (Figure 4B). To comprehensively evaluate the severity of these symptoms, Psoriasis Area and Severity Index (PASI) scores were calculated. We observed that redness, thickness and scaling of the skin began to manifest two days after IMQ treatment in WT mice, whereas CD169-DTR mice consistently exhibited lower PASI scores from day 2 to day 7 (Figure 4C). Both groups of mice experienced comparable body weight loss after IMQ application (Figure 4D). Histological analysis further revealed a remarkable increase in epidermal thickness and the number of Ki-67 positive keratinocytes in WT mice after IMQ treatment. In contrast, these phenotypes were significantly reduced in CD169-DTR mice (Figure 4E–H). Collectively, these findings provide compelling evidence that the depletion of CD169^+^ macrophages can alleviate IMQ-induced psoriasis-like symptoms.

### 2.3. Depletion of CD169^+^ Macrophages Suppresses the Production of Psoriasis Related Cytokines and Reduces the Frequency of Th17 Cells in the Skin

**Figure 5:**
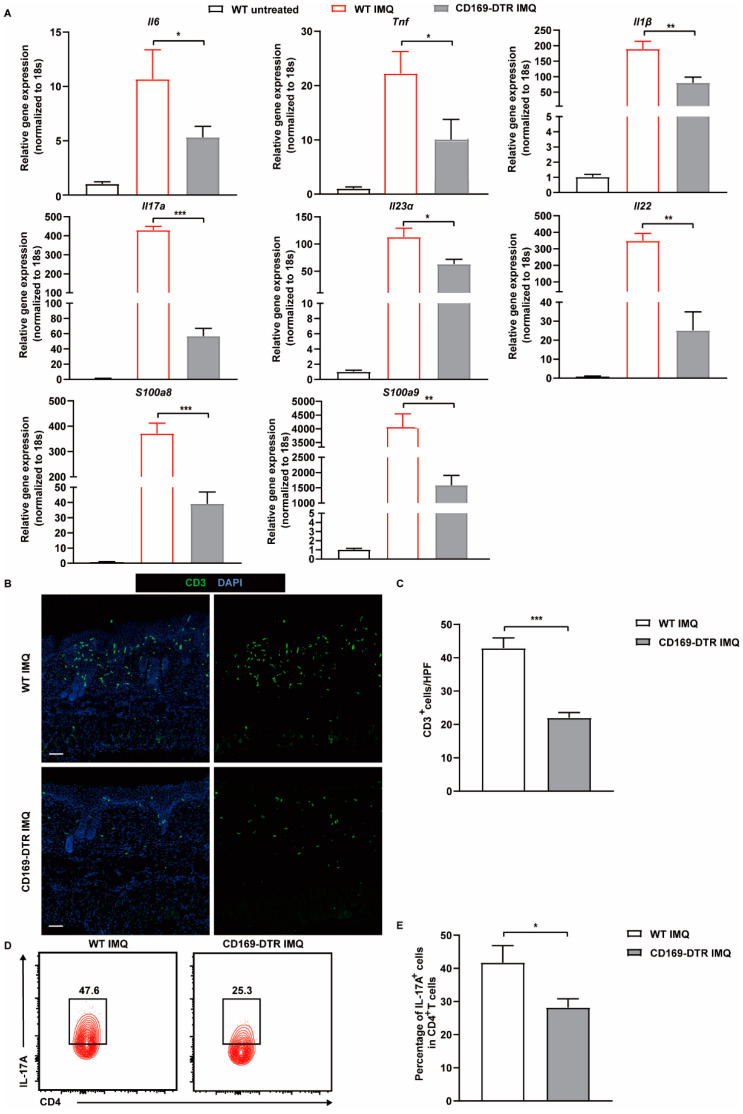
The expression of proinflammatory cytokines and the number of Th17 cells in the skin are reduced in IMQ-treated CD169-DTR mice. (**A**) *Il6*, *Tnf*, *Il1β*, *Il23α*, *Il22*, *Il17a*, *S100a8*, *S100a9* mRNA levels in untreated WT, IMQ-treated WT and CD169-DTR mice detected by qRT-PCR analysis on day 7 (*n* = 3–4). (**B**) Representative immunofluorescence staining of CD3 in the skin of IMQ-treated WT and CD169-DTR mice on day 7 (green: CD3, blue: DAPI, *n* = 3). Scale bar: 100 μm. (**C**) Quantification of CD3^+^ T cells in (**B**). (**D**) Representative flow cytometry plots show the percentage of IL-17A^+^ T cells in the skin of IMQ-treated WT and CD169-DTR mice. The cells were gated from CD3^+^CD4^+^ cells. (**E**) Quantification of the percentage of IL-17A^+^ Th17 cells in CD4^+^ T cells detected by flow cytometry on day 7 (*n* = 3). Data represent at least two independent experiments. *** *p* < 0.001, ** *p* < 0.01, * *p* < 0.05 (Student’s *t*-test).

The pathogenesis of psoriasis is orchestrated by the intricate interplay among diverse cell types and inflammatory cytokines [30]. Given the pivotal roles of pro-inflammatory cytokines such as IL-6, TNF-α, IL-1β, and Th17-associated cytokines, in psoriasis development, we compared their expression levels in IMQ-treated WT and CD169-DTR mice. We performed quantitative real-time reverse transcription PCR (qRT-PCR) on skin samples collected from both groups on day 7. As expected, the expression of pro-inflammatory cytokines (*Il6*, *Tnf*, *Il1β*, *Il23α*, *Il17a*, and *Il22*) was increased following IMQ treatment. However, this trend was significantly attenuated in IMQ-treated CD169-DTR mice, with a remarkable 10-fold reduction observed in *Il17a* expression. Additionally, the expression of alarmins *S100a8* and *S100a9*, which have been considered as reliable biomarkers for monitoring inflammatory disease activity in psoriasis, was also decreased (Figure 5A). IL-17A, the inflammatory cytokine with the most significant reduction following CD169^+^ macrophage depletion, is primarily secreted by T cells, specifically Th17 cells and γδ T cells. Hence, we initially assessed T cell infiltration via immunofluorescence staining, which revealed a lower abundance of CD3^+^ T cells in the skin of IMQ-treated CD169-DTR mice (Figure 5B,C). Further flow cytometric analysis showed that the depletion of CD169^+^ macrophages led to a decrease in the percentage of Th17 cells (CD4^+^IL-17A^+^), dropping from 39.2% (±2.92%) to 27.9% (±1.51%) (Figure 5D,E). This result aligns with the considerably diminished RNA expression levels of *Il23α*, *Il17a*, and *Il22* in the skin lesions of CD169-DTR mice. In conclusion, CD169^+^ macrophages play a crucial role in modulating the production of psoriasis-related cytokines and the frequency of Th17 cells.

### 2.4. CD169^+^ Skin Macrophages Exhibit Unique Gene Expression Profiles upon IMQ Stimulation

**Figure 6:**
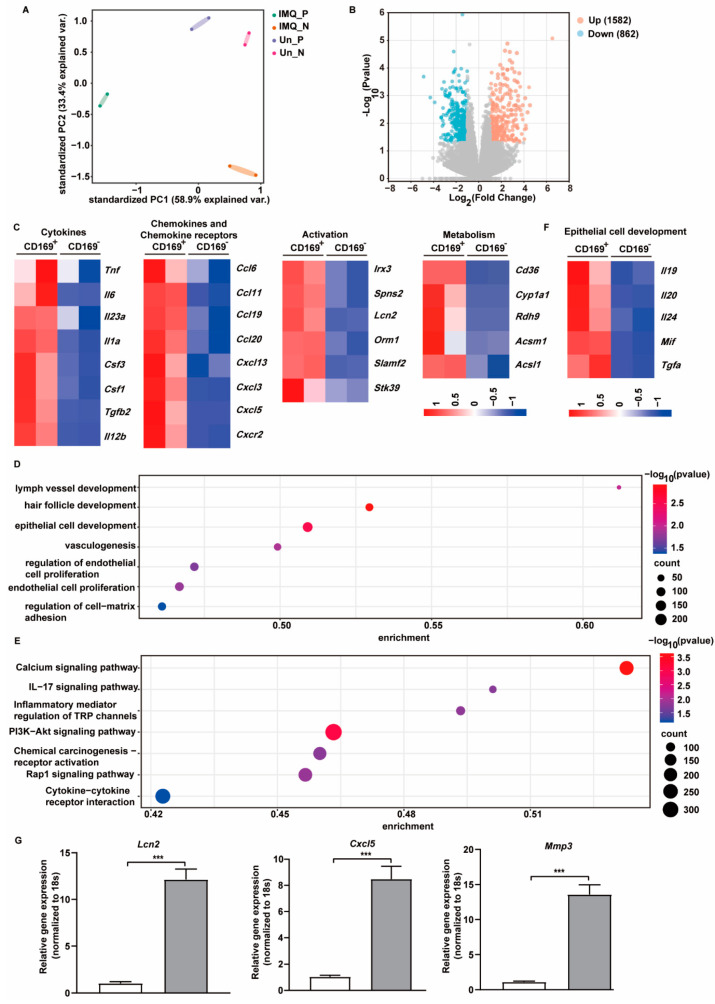
The unique transcriptional profile of CD169^+^ skin macrophages after IMQ treatment. CD169^+^ and CD169^−^ macrophages were isolated from untreated or IMQ-treated WT mice skin and subjected to RNA-seq. (**A**) PCA plot of all the samples. (**B**) Volcano plot of DEGs in IMQ-treated CD169^+^ macrophages compared with IMQ-treated CD169^−^ macrophages. (**C**) Heatmaps represent expression of upregulated DEGs related to cytokines, chemokines and chemokine receptors, macrophage activation and metabolism. (**D**,**E**) Gene ontology (GO) analysis and Kyoto encyclopedia of genes and genomes (KEGG) pathway analysis of upregulated DEGs in IMQ-treated CD169^+^ macrophages. (**F**) Heatmap represents expression of upregulated DEGs related to epithelial cell development. (**G**) *Mmp3*, *Lcn2*, *Cxcl5* mRNA levels in IMQ-treated CD169^+^ and CD169^−^ skin macrophages detected by qRT-PCR (*n* = 3). The qRT-PCR results represent at least two independent experiments. *** *p* < 0.001 (Student’s *t*-test).

To decipher the molecular mechanisms underlying the impact of CD169^+^ skin macrophages on inflammation and psoriasis, we sorted CD169^+^ and CD169^−^ macrophages from the skin of untreated or IMQ-treated WT mice and performed RNA-seq analysis. Unsupervised principal component analysis (PCA) was used to evaluate the association between the samples, revealing that the global gene expression profiles of CD169^+^ (Un_P) and CD169^−^ (Un_N) macrophages isolated from normal skin exhibited a higher degree of similarity to each other. In sharp contrast, these two populations of macrophages became distinct from each other following IMQ treatment, indicating a substantial difference between the two groups (IMQ_P, IMQ_N) (Figure 6A). Subsequently, we conducted differential expression analysis to compare CD169^+^ and CD169^−^ macrophages after IMQ stimulation. We identified 1582 upregulated and 862 downregulated genes in CD169^+^ macrophages (Figure 6B).

Given the amelioration of IMQ-induced psoriasis-like symptoms upon depletion of CD169^+^ macrophages, our attention focused on the differentially expressed genes (DEGs) that were upregulated in the IMQ_P group compared with the IMQ_N group. We first examined the cytokines and chemokines in these DEGs. The heatmaps revealed an increase in several classic proinflammatory cytokines such as *Tnf*, *Il6*, *Il1a*, *Il23α* and a small number of chemokines, including *Ccl19*, *Ccl20*, *Cxcl13*, and *Cxcl5*, which are typically elevated in psoriasis patients, in CD169^+^ macrophages. Notably, we observed an upregulation of genes related to cell activation, including *Irx3*, *Spns2*, *Lcn2* and genes related to cell metabolism such as *Cd36*, and *Cyp1a1*, in CD169^+^ macrophages following IMQ treatment, indicating a more active state of CD169^+^ macrophages in psoriasis compared to CD169^−^ macrophages (Figure 6C).

To gain further insight into the biological function and potential signaling pathways associated with these DEGs, we conducted GO and KEGG analyses. The enriched biological processes included lymph vessel development, hair follicle development, epithelial cell development, vasculogenesis and regulation of endothelial cell proliferation, suggesting a close contact between CD169^+^ macrophages and surrounding epithelial or endothelial cells (Figure 6D). KEGG pathway analysis revealed the association of these genes with pathways such as IL-17 signaling, PI3K-Akt signaling, Rap1 signaling and cytokine–cytokine receptor interaction (Figure 6E). Among these DEGs, genes related to epithelial cell development were displayed by heatmap (Figure 6F). The qRT-PCR result confirms that three highly expressed DEGs (*Mmp3, Cxcl5, Lcn2*) were upregulated in IMQ-treated CD169^+^ macrophages, which is consistent with the RNA-seq findings. Specifically, *Mmp3* and *Lcn2* displayed more than a 10-fold increase, while *Cxcl5* exhibited an approximately 8-fold increase (Figure 6G). These results suggest that CD169^+^ and CD169^−^ skin macrophages exhibit distinct responses to IMQ stimulation, and that the unique upregulated genes in IMQ-treated CD169^+^ skin macrophages may be the key to their function in psoriasis.

### 2.5. CD169^+^ Skin Macrophages Promote Th17 Cell Differentiation Ex Vivo

**Figure 7:**
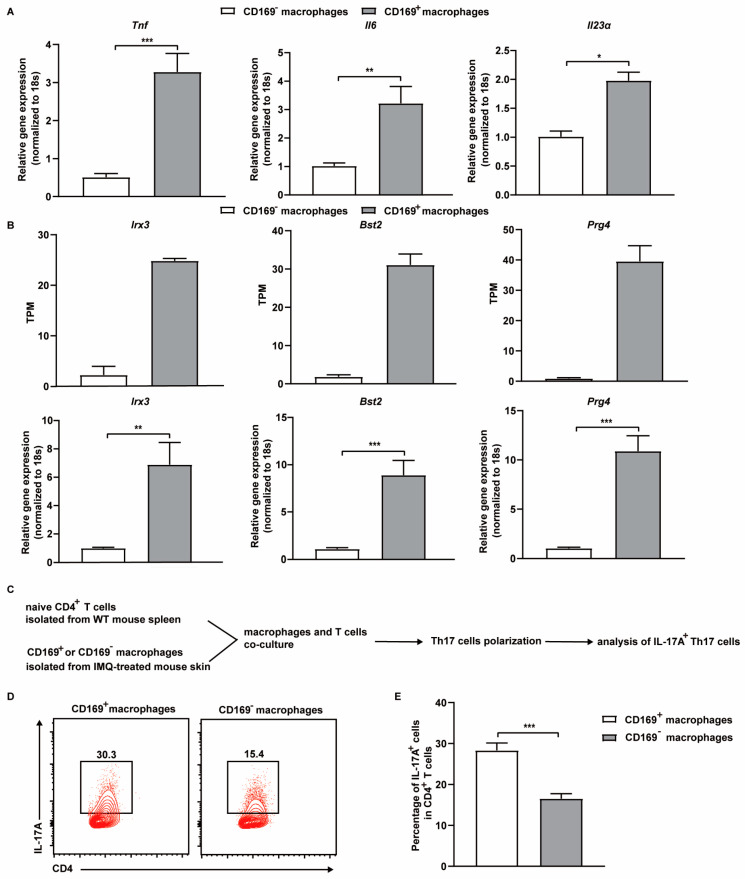
IMQ-treated CD169^+^ skin macrophages promote in vitro differentiation of Th17 cells. (**A**) The expression levels of *Tnf*, *Il6*, and *Il23α* in IMQ-treated CD169^+^ and CD169^−^ skin macrophages detected by qRT-PCR (*n* = 3). (**B**) The expression levels of *Irx3*, *Bts2*, *Prg4* in IMQ-treated CD169^+^ and CD169^−^ skin macrophages detected by RNA-seq and qRT-PCR (*n* = 3 for qRT-PCR). (**C**) Schematic diagram of the co-culture experiment. (**D**) Representative flow cytometry plots show the percentage of IL-17A^+^ T cells in the co-culture system (*n* = 3). (**E**) Quantification of the percentage of IL-17A^+^ Th17 cells in CD4^+^ T cells in the co-culture experiment (*n* = 3). Data (except for RNA-seq result) represent at least two independent experiments. *** *p* < 0.001, ** *p* < 0.01, * *p* < 0.05 (Student’s *t*-test).

The previous findings of reduced Th17 cells in the skin of IMQ-treated CD169-DTR mice raise the possibility that CD169^+^ macrophages could directly participate in regulating Th17 cell differentiation. Activated macrophages can produce cytokines such as TNF-α, IL-6, and IL-23, which are critical for the differentiation of Th17 cells, in psoriasis lesions [31]. Therefore, we first compared the expression level of these cytokines between IMQ-treated CD169^+^ and CD169^−^ skin macrophages using qRT-PCR. Both *Il6* and *Tnf* showed upregulation of approximately 3–4 fold in CD169^+^ macrophages, while *Il23α* exhibited a 2-fold increase. Furthermore, the RNA-seq and the qRT-PCR results show the upregulation of genes (*Irx3*, *Bst2*, *Prg4*) involved in macrophage activation in CD169^+^ skin macrophages (Figure 7A,B). Next, to directly test our hypothesis, we established a coculture system using CD169^+^ and CD169^−^ skin macrophages isolated from IMQ-treated mice. The macrophages were cultured with naïve T cells at a 1:1 ratio under modified Th17-driving conditions, which included sub-optimal concentrations of exogenous IL-6, IL-23, and TGF-β. Strikingly, the percentage of Th17 cells was 2-fold higher in the coculture system of CD169^+^ macrophages than CD169^−^ macrophages (Figure 7C–E). These findings collectively demonstrate that CD169^+^ skin macrophages are more capable of promoting Th17 cell differentiation in psoriasis.

## 3. Discussion

The skin, the outermost layer of our body, is constantly exposed to external stimuli. As an immune organ, the skin serves as an effective barrier protecting the body against pathogenic and environmental insults [32,33]. Various types of innate and adaptive immune cells inhabit the skin, with macrophages and dendritic cells being the predominant populations [34]. Extensive research indicates that macrophages participate in hair follicle regeneration, wound healing and the stress response, highlighting their indispensable contribution to the maintenance of skin homeostasis and integrity [35]. In this study, we demonstrated the crucial involvement of CD169^+^ skin macrophages in the development of psoriasis. Depletion of CD169^+^ skin macrophages, which showed a distinct gene expression pattern upon IMQ stimulation, significantly relieved the symptoms of psoriasis by impairing the production of proinflammatory cytokines and by affecting the frequency of Th17 cells.

Dermal macrophages consist of several heterogeneous subpopulations originating from distinct sources. Some dermal macrophages predominantly originate from progenitors present in the dermis prenatally, such as sensory-nerve-associated macrophages. Conversely, other subpopulations are gradually replenished by monocyte-derived macrophages after birth [15,36]. Previous research has reported that most CD169^+^ skin macrophages are bone marrow-derived and exhibit low expression of CX3CR1, indicating that they are replenished by bone marrow-derived monocytes in steady state [25]. In our study, we observed a pronounced increase in the number of CD169^+^ macrophages during the induction of psoriasis in mice. Interestingly, these macrophages did not seem to possess the capacity for self-renewal, as evidenced by their lack of expression of the cell proliferation marker Ki-67. Instead, we found that a portion of CD169^+^ skin macrophages was labeled with tdTomato during short-term tamoxifen treatment in *Ms4a3^CreERT2^*-*Rosa26^tdTomato^* mice. These findings convincingly indicate that monocytes are recruited and differentiate into CD169^+^ skin macrophages, which can promote the development of psoriasis.

Traditionally, activated macrophages can be categorized into two main phenotypes: M1 macrophages, characterized by vigorous secretion of pro-inflammatory cytokines such as IL-6, TNF-α, and IL-1β, and M2 macrophages, which release anti-inflammatory mediators and matrix metalloproteinases [37]. Intriguingly, our RNA-seq results demonstrate that CD169^+^ skin macrophages displayed a unique gene expression profile, different from the classic M1/M2 macrophage polarization, upon IMQ stimulation. Specifically, CD169^+^ skin macrophages from IMQ-treated mice showed upregulation of M1-related inflammatory cytokines and chemokines, including *Il6*, *Tnf*, *Il23α*, *Cxcl3,* and *Cxcl5,* as well as M2-related genes *Mmp3* and *Mmp9*. Similar phenomena have also been reported in alveolar macrophages, which also express CD169 [38,39]. During the course of pulmonary fibrosis, alveolar macrophages upregulated both M1 and M2 genes in response to bleomycin without an overwhelming shift in gene expression toward an M1 or M2 phenotype [39]. Although M1-like and M2-like macrophages, which share some characteristics with in vitro generated M1/M2 macrophages, have been reported in in vivo settings, the intricacy of the in vivo environment renders macrophage polarization more complicated than a simplistic M1/M2 classification, as we have observed in CD169^+^ skin macrophages. Nevertheless, it is possible that additional subpopulations could exist within CD169^+^ skin macrophages, which merits further examination through single-cell RNA-seq analysis.

Macrophages and dendritic cells have been identified as the main source of IL-23 in human psoriasis lesions through immunohistochemistry staining and single cell sequencing [40]. Moreover, CD169^+^ macrophages have been reported to mediate the recruitment and activation of γδ T cells through IL-23 signaling during Staphylococcus aureus skin infection [25]. Remarkably, we found that CD169^+^ macrophages secrete much more proinflammatory cytokines and are superior in promoting Th17 differentiation in an in vitro system compared to CD169^−^ macrophages, which demonstrate that CD169^+^ skin macrophages constitute a distinct population with unique advantages in facilitating the development of psoriasis. Thus, it is intriguing to understand the precise mechanism underlying the enhanced cytokine production in CD169^+^ skin macrophages, and our RNA-seq data provide certain clues. Activated macrophages typically exhibit alterations in cellular metabolism, which play a crucial role in influencing immune cell functions, including cytokine production [41]. Genes associated with cell metabolism such as *Cd36* were upregulated in CD169^+^ macrophages according to our sequencing data. As shown in Roy L. Silverstein’s study, CD36 signaling can facilitate NF-κB activation and proinflammatory status in macrophages by regulating mitochondrial metabolism [42]. Additionally, some transcription factors that have been reported to regulate proinflammatory gene expression in macrophages, such as *Irx3* [43], were also upregulated in CD169^+^ macrophages. These changes may account for the enhanced cytokine production in CD169^+^ skin macrophages.

In addition to regulating the function of immune cells by secreting cytokines and chemokines, macrophages can directly influence epithelial cells, endothelial cells and fibroblasts in the skin. For example, macrophages can release growth factors, including epidermal growth factor, keratinocyte growth factor, TGF-α, and VEGF, to stimulate fibroblast and keratinocyte proliferation as well as to promote angiogenesis during skin wound healing [44,45]. Notably, CD169^+^ skin macrophages may exert similar effects during psoriasis development. Firstly, the GO analyses of the RNA-seq results reveal the enrichment of genes associated with epithelial cell development, regulation of endothelial cell proliferation and vasculogenesis, such as *Il19*, *Il20*, and *Mif*, in CD169^+^ macrophages compared with CD169^−^ macrophages. Importantly, we observed that CD169^+^ macrophages appeared near the epidermis, where keratinocytes are located, following IMQ application, which increases the likelihood of their spatial interaction with keratinocytes. All of this evidence strongly suggests that CD169^+^ skin macrophages may directly interact with and impact epithelial cells during psoriasis, which could be further explored in the future.

Nevertheless, our study has certain limitations. Firstly, it was conducted using mouse models, which necessitates the validation of our findings in clinical samples to determine whether CD169^+^ skin macrophages exhibit similar functions during psoriasis development in humans. Furthermore, while our study primarily focused on the impact of CD169^+^ skin macrophages on Th17 cells, it is possible that other immune cells may also be influenced by CD169^+^ skin macrophages due to their heightened expression of proinflammatory cytokines and chemokines when compared with CD169^−^ skin macrophages following IMQ treatment. Additionally, CD169^+^ skin macrophages isolated from IMQ-induced psoriasis skin lesions exhibited upregulation of several genes associated with cell activation or metabolism. However, the specific genes that are key to their unique functions remain undefined and warrant further investigation in future studies.

In summary, our work has uncovered the critical functions performed by CD169^+^ skin macrophages in the development of psoriasis. These findings may deepen our understanding of skin macrophage heterogeneity and provide implications for psoriasis treatment.

## 4. Materials and Methods

### 4.1. Mice

Wild type C57BL/6 mice were purchased from the Shanghai SLAC Laboratory Animal Center. CD169-DTR mice were developed by Dr. Makoto Tanaka and imported from the RIKEN BioResource Center (Wako, Japan). *Ms4a3^CreERT2^*-*Rosa26^tdTomato^* mice were obtained from Dr. Zhaoyuan Liu (Shanghai Institute of Immunology, Shanghai Jiao Tong University School of Medicine, China). All mice used in this study were maintained in a SPF facility at the Shanghai Jiao Tong University School of Medicine Animal Resource Center. All animal experiments were conducted in accordance with the protocols approved by the Institutional Animal care and Use Committee of Shanghai Jiao Tong University School of Medicine (JUMC2023-152-A).

### 4.2. IMQ-Induced Mouse Psoriasis Model and DT Injection

For the IMQ-induced psoriasis mouse model, WT and CD169-DTR mice were topically treated with a daily dose of 60 mg of 5% IMQ cream (MedShine, Chengdu, China) on the shaved back skin for six consecutive days [27]. For CD169^+^ macrophages depletion, CD169-DTR mice were injected intraperitoneally with DT (20 ng/g body weight) every other day until mice were sacrificed. 

### 4.3. Tamoxifen Administration

Tamoxifen (Sigma, St. Louis, MO, USA) was dissolved in corn oil at a concentration of 20 mg/mL by shaking overnight at 37 °C. *Ms4a3^CreERT2^*-*Rosa26^tdTomato^* mice were injected intraperitoneally with tamoxifen (200 mg/kg body weight) once a day for three consecutive days and then imiquimod was used to induce a psoriasis model as previously described [28]. During IMQ treatment, tamoxifen was administered via intraperitoneal injection every other day until mice were sacrificed on the sixth day. 

### 4.4. Psoriasis Area Severity Index (PASI) and Bodyweight 

The clinical Psoriasis Area and Severity Index (PASI) was used to assess the severity of psoriasis symptoms. Redness, thickness and scaling were scored independently on a scale from 0 to 4 (0—none, 1—slight, 2—moderate, 3—marked, 4—very marked) for 7 consecutive days. The score of the three indices was summed up as the total score (0−12), which represented the severity of the psoriasis symptoms [46]. The bodyweight of each mouse was recorded daily for one week.

### 4.5. Flow Cytometry

To obtain single-cell suspensions, 4 cm^2^ of back skin tissue was mechanically disrupted and digested with 2 mg/mL Dispase II (Roche, Basel, Switzerland) for 1 h at 37 °C. The fragments were further digested with 1 mg/mL Collagenase P (Sigma) and 0.1 mg/mL DNase I (Roche) in DMEM containing 1% FBS for 1.5 h at 37 °C. After filtrating through a 70 μm cell strainer, the cells were resuspended in PBS containing 2% FBS [47]. For surface staining, cell suspensions were stained with LIVE/DEAD fixable dead cell stain (Invitrogen, Carlsbad, CA, USA) for 10 min at 4 °C, then blocked with anti-CD16/32 (clone 2.4G2, BD Biosciences) for 10 min, followed by staining with fluorochrome-conjugated antibodies for 30 min at 4 °C. To stain Th17 cells, the cell suspensions were first stimulated with a cell stimulation cocktail with Brefeldin A (Biolegend, San Diego, CA, USA) for 4 h, then surface stained, fixed, permeabilized and stained with anti-IL-17A antibody. The following antibodies were used: anti-CD169 (SER4, eBioscience, San Diego, CA, USA), anti-F4/80 (BM8, Biolegend), anti-CD11b (M1/70, Biolegend), anti-CD11c (N418, Biolegend), anti-CD45 (QA17A26, Biolegend), anti-Ly6G (S19018G, Biolegend), anti-Ly6C (HK1.4, Biolegend), anti-CD3 (17A2, Biolegend), anti-CD4 (RM4-5, Biolegend), anti-IL-17A (HA, Biolegend), and Rat IgG2a kappa Isotype Control (eBioscience). All samples were acquired with LSRFortessa X-20 (BD biosciences, San Jose, CA, USA), and the results were analyzed using FlowJo software (version 10.8, BD biosciences, San Jose, CA, USA).

### 4.6. Skin Macrophages Isolation

For macrophages isolation, single-cell suspensions were prepared as previously described. The cell suspensions were resuspended in DMEM, then carefully loaded on top of 2 mL lymphocyte separation medium (Dakewe Biotech Co., Shenzhen, China) and centrifuged at 1800 rpm for 20 min. The cells in the middle layer were collected and stained with fluorochrome-conjugated antibodies. Skin CD169^+^ and CD169^−^ macrophages were sorted by FACSAria III (BD biosciences).

### 4.7. Immunofluorescence Staining

Skin samples were fixated overnight in 1% PFA, dehydrated in 30% sucrose, embedded in OCT and cut into 20 μm thick sections. The sections were permeabilized with cold methanol for 20 min and blocked with blocking buffer (0.1 M Tris-HCl, 0.3% Triton X-100, 1% FBS, 1% BSA and 1% normal mouse serum in PBS) for 1 h at 25 °C. The sections were then incubated with antibodies diluted in blocking buffer (1:200) for 3 h at room temperature. After washing three times with PBS, the sections were stained with DAPI and mounted with Fluoromount-G (Southern Biotech, Birmingham, AL, USA). Images were observed with Leica TCS SP8 confocal microscopy (Leica, Wetzlar, Germany) and processed using Imaris (version 9.7.0, Bitplane, Oxford, UK).

### 4.8. Hematoxylin and Eosin Staining

Skin samples were fixed in 4% PFA and embedded in paraffin. These samples were then cut into 5 μm sections and stained with hematoxylin and eosin. Images were captured with NanoZoomer S360 and then processed with NDP.view 2.0 (Hamamatsu photonics K.K., Hamamatsu, Japan).

### 4.9. In Vitro Th17 Cell Differentiation 

Naïve CD4^+^ T cells were isolated from the spleens of WT mice and co-cultured with CD169^+^ or CD169^−^ macrophages isolated from IMQ-induced psoriasis-like skin lesions at a ratio of 1:1 for 3 days in RPMI 1640 containing 1 ng/mL TGF-β, 10 ng/mL IL-6, 10 ng/mL IL-23, 5 μg/mL anti-IL-4 antibody and 5 μg/mL anti-IFNγ antibody. On day 4, the percentage of CD4^+^IL-17A^+^ cells was analyzed by flow cytometry.

### 4.10. RNA Extraction and Quantitative Real-Time Reverse Transcription PCR (qRT-PCR)

Total RNA was extracted by TRIzol reagent (Life Technologies, Gaithersburg, MD, USA) and transcribed into cDNA by Hifair II 1st Strand cDNA Synthesis SuperMix for qRT-PCR (Yeasen, Shanghai, China). qRT-PCR was performed using SYBR Green Real-time PCR Master Mix (TOYOBO, Osaka, Japan) and ViiA7 Real-Time PCR System (Applied Biosystems, Waltham, MA, USA). Relative mRNA levels were normalized to the *18s* and calculated using the 2^−ΔΔCt^ method. Oligonucleotide sequences of qRT-PCR primers are listed in Table S1.

### 4.11. RNA-Seq

Total RNA was extracted from skin CD169^+^ or CD169^−^ macrophages by TRIzol reagent (Life Technologies). RNA integrity was analyzed by Agilent 2100 Bioanalyzer (Agilent Technologies, Santa Clara, CA, USA). RNA-seq libraries were prepared with Illumina TruSeq RNA Library Preparation Kit v2 and sequenced (PE, 2 × 150 bp) on an Illumina HiSeq 2000. All the reads were mapped to the mouse reference genome (GRCm39) using STAR aligner (v2.5.0c). Differential gene expression (fold change ≥ |1.5|, *p*_adjusted < 0.05) were calculated using DESeq2 (v3.0). KEGG pathway analysis, gene ontology (GO) analysis, heatmap and volcano plots were performed using R packages.

### 4.12. Statistical Analysis

Statistical analyses were performed using GraphPad Prism 8.0 (GraphPad Software, San Diego, CA, USA). All data are expressed as means ± SEM and data values were compared with unpaired Student’s *t*-test. The difference was statistically significant when *p* < 0.05.

## 5. Conclusions

In this study, we have demonstrated the crucial role of CD169^+^ skin macrophages, predominantly located in the lower dermis and dermal white adipose tissue, in the pathogenesis of IMQ-induced psoriasis-like skin disease. Throughout the progression of psoriasis, the population of CD169^+^ skin macrophages gradually increased and began to appear closer to the epidermis, where keratinocytes reside. Importantly, selective depletion of CD169^+^ macrophages led to the improvement of psoriasis symptoms, including erythema, scaling, and skin thickening. This was accompanied by reduced levels of proinflammatory cytokines and a diminished proportion of Th17 cells in the skin lesions. Additionally, we identified a distinctive gene signature specific to CD169^+^ skin macrophages following IMQ treatment, and this particular subpopulation of macrophages exhibited an enhanced capability to induce Th17 cell differentiation in vitro. In future investigations, we will strive to unravel the precise molecular mechanisms underlying the activation of CD169^+^ macrophages during psoriasis development.

## Data Availability

The data presented in this study are available in the article.

## Supplementary Materials

The following supporting information can be downloaded at: https://www.mdpi.com/article/10.3390/ijms25115705/s1.
