# Supplementary material for: CD169+ Skin Macrophages Function as a Specialized Subpopulation in Promoting Psoriasis-like Skin Disease in Mice
Source: Int J Mol Sci. 2024 May 24;25(11):5705. doi: 10.3390/ijms25115705 (PMC11171985; doi:10.3390/ijms25115705)
Supplement: Supplementary file 1 [file ijms-25-05705-s001.zip › ijms-2999116-supplementary.pdf]

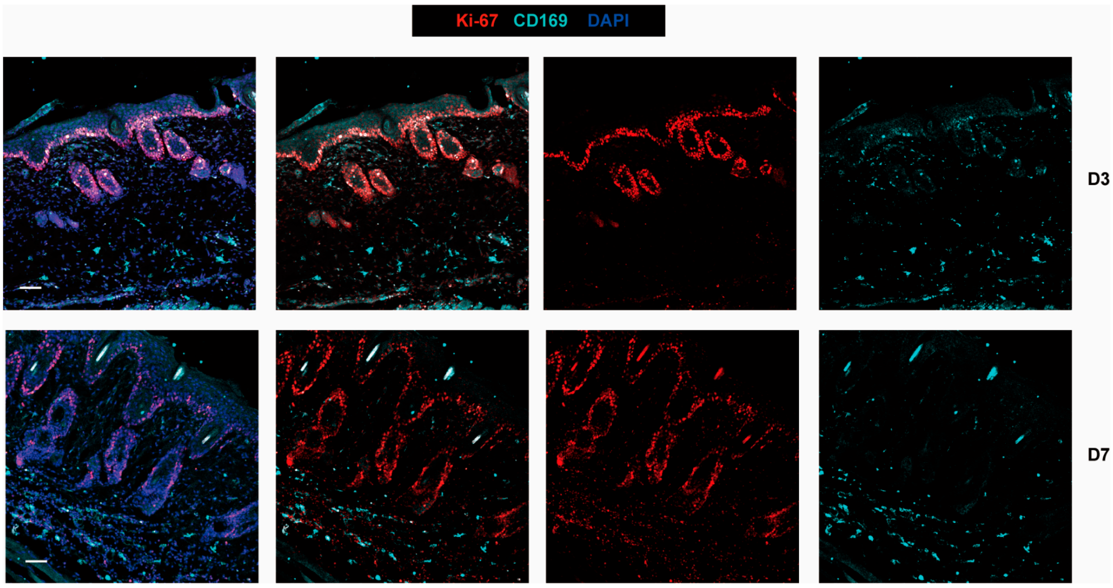

Figure S1. CD169⁺ macrophages do not express Ki-67 in psoriasis lesions. Representative immunofluorescence staining of CD169 and cell proliferation marker Ki-67 in IMQ-treated WT mice skin on day 3 and day 5 (red: Ki-67, cyan: CD169, blue: DAPI, n=3). Scale bar: 100 μm.
